# Evaluating global research trends in special needs dentistry: A systematic bibliometrix analysis

**DOI:** 10.1002/cre2.896

**Published:** 2024-06-16

**Authors:** Nigashiny Senthilvadevel, Jimmy Ky, Matthew Ng, Tong Zhao, Massimo Aria, Luca D'Aniello, Mathew A. W. Lim, Federica Canfora, Giulio Fortuna, Michael McCullough, Tami Yap, Rita Paolini, Antonio Celentano

**Affiliations:** ^1^ Melbourne Dental School The University of Melbourne Carlton Victoria Australia; ^2^ Department of Economics and Statistics University of Naples Federico II Naples Italy; ^3^ Department of Social Sciences University of Naples Federico II Naples Italy; ^4^ Department of Neuroscience, Reproductive Sciences and Dentistry University of Naples Federico II Naples Italy; ^5^ Department of Oral Medicine, Glasgow Dental Hospital and School University of Glasgow Glasgow UK

**Keywords:** bibliometric, special needs dentistry, systematic

## Abstract

**Objectives:**

Special needs dentistry (SND) is a vast and fragmented field of study. This comprehensive bibliometric analysis aimed to evaluate the scope of SND, including the existing knowledge base, distribution structure, quantitative relationships, and research trends.

**Material and Methods:**

A systematic search was conducted on March 10, 2022, using the Web of Science Core Collection database, covering the period from 1985 to 2021, focusing on studies reporting on special needs populations in a dentally relevant context. Records were title‐screened and analyzed for key bibliometric indicators.

**Results:**

Among 48,374 articles, 13,869 underwent bibliometric analysis. Peak SND research occurred during 1985–1997. United States led in productivity, trailed by Brazil and Japan. University of Sao Paulo excelled in Brazil, University of Washington and University of North Carolina in the United States. The *Journal of Dental Research* was the most productive source of research and also had the highest number of citations, followed by *Community Dentistry and Oral Epidemiology*. Keyword analysis revealed that “elderly”, “caries”, and “epidemiology” were the most commonly used author keywords.

**Conclusions:**

This study represents the first bibliometric analysis of SND literature. It emphasizes the need for increased collaboration between institutions and authors. Furthermore, it suggests focusing on research input from non‐dental disciplines and populations with rarer intellectual or developmental conditions.

## 1 INTRODUCTION

Special needs dentistry (SND) has emerged as a discipline in light of the unmet needs of many individuals with disabilities and complex health issues. The Dental Board of Australia defines SND as a discipline that is concerned with the oral health of people with an intellectual disability, medical, physical, or psychiatric conditions (including patients under geriatrics and those with limited access) and those requiring special dental methods or techniques (Dental Board of Australia ‐ Registration Standards Internet, 2023).

Despite the considerable negligence by the traditional care schemes, the population of people requiring SND care is vast and varied. According to the Australian Institute of Health and Welfare, in 2022, one in six Australians, or approximately 4.4 million people, had disabilities. Among them, 32% suffered from severe disabilities where daily self‐care, communication, and mobility are affected. In addition, there are 4.2 million people over 65 years old in Australia, who may also benefit from some specialist care (People with disability in Australia, About Internet, 2023). These individuals experience higher levels of oral disease and the greater unmet dental need than the rest of the population, often associated with concerns regarding cooperation, cognitive capacity, and communication (Waldman & Perlman, 2010). Perceived lack of formal education in SND, financial barriers, the assumed intrusions into practice dynamics and the increased complexities to care are often highlighted by general dental clinicians in their reluctance to treat patients with special needs indicating the need for comprehensive research in this field (Derbi & Borromeo, 2016).

The field of study of SND, however, is voluminous and fragmented. To appreciate the breadth of current research and highlight unexplored areas, the analysis of research trends is crucial. Open‐source tools for the comprehensive analysis on extant research, such as bibliometrics, can help apply statistical methods to the study of bibliographic data (Roy and Basak, 2013) *Bibliometrix R Package* is one such software that facilitates the importing of data from distinguished bibliographic databases and inferring on productivity, impact, growth and development of the existing research field (Aria & Cuccurullo, 2017). *Biblioshiny*, a noncoding web interface of *Bibliometrix* and still encompassing the main analytical features of the parent program, is efficient, accessible, and can ultimately provide invaluable insight to the everyday researcher.

This study aims to evaluate the scope of SND, in terms of the existing knowledge base, distribution structure, quantitative relationships, and research trends, since its conception through a comprehensive bibliometric analysis.

## 2 MATERIALS AND METHODS

Our review followed the Preferred Reporting Items for Systematic Reviews and Meta‐Analysis (PRISMA) guidelines (Moher et al., 2015). The search was conducted on the Web of Science Core Collection database on March 10, 2022. Searches focused on studies based on special‐needs populations with a dentally relevant context. To identify such publications, we incorporated the use of MeSH terms such as “intellectual disab*”, “physical disab*”, “syndrome”, “special need*” among others in a single search string separated by the OR operator. The full search strategy was developed in collaboration with a tertiary librarian and can be found in the Appendices.

### 2.1 Inclusion and exclusion criteria

Inclusion criteria included studies published in the English language. Eligible document types included original articles, conference abstracts, reviews, proceedings papers, and book chapters. In‐vitro and animal studies were not eligible for inclusion.

Categorizations of special needs populations eligible for inclusion were based on the World Health Organization's International Classification of Functioning, Disability and Health for both adults and children (World Health Organization, 2001, 2007). This framework uses disability as an umbrella term to include bodily impairments, activity limitations, or participation restrictions in the context of one's environment. In alignment with the definition of SND, the authors also included populations whom they believed to require specially considered treatment planning, specialized dental methods and techniques, such as migrant, homeless, substance misuse, or incarcerated patient populations. The authors had also included studies with patient populations based in countries with poverty and low income or lower‐middle income economies, according to the World Bank Country Classification by income level: 2022–2023 (World Bank Country and Lending Groups – World Bank Data Help Desk Internet, 2023). Countries defined as having a low or lower‐middle income economies were defined as having a gross national income per capita of $1085 or less in 2021, and between $1086 and $4255, respectively.

### 2.2 Data extraction

Retrieved records were divided among four members of the research team, which were divided into two groups (JK, NS and TZ, MN). The groups were calibrated before the final search via repeated calibration trials. Calibration trials consisted of a random sample of 50 studies which were title‐screened by each author within each team until the Cohen's Kappa value met the predefined threshold level of 0.75. Subsequent to this condition being met, the final search was conducted. Following duplicate removal, records were title‐screened using Covidence (Covidence Systematic Review Software) according to the eligibility criteria. Any conflicts which arose during the calibration or initial screening stage were resolved by discussion.

Eligible studies were imported into the biblioshiny web‐app for bibliometrix analysis (Aria & Cuccurullo, 2017). The software was used to extract and analyze relevant bibliometric indicators: number of articles, source, keywords assigned by the system and authors, average citations per article, number of authors, number of author appearances, authors per article, authors of single‐authored and multi‐authored articles, co‐authors per article, annual scientific production and citations, most productive authors, author affiliations, author indices (h‐index, g‐index, m‐index), most cited articles and cited references, most productive countries, institutions or departments, as well as the top relevant keywords. The author level metrics Hirsch index (h‐index) and the g‐index were used to quantify the number of publications and number of citations per publication per author.

### 2.3 Statistical analysis

The collected data have been processed by bibliometrix (Ver 4.0.1) to generate descriptive analyses, statistical plots or graphs, and scientific maps.

## 3 RESULTS

Supporting Information S1: Appendix Figure 1 outlines the study selection process using a PRISMA flow diagram. The final search across the Web of Science database returned 48,374 records. After removal of 179 duplicates, 48,195 articles were selected for title screening. Of the eligible articles, 34,326 articles were excluded based on the inclusion criteria, resulting in a final selection of 13,869 articles to proceed to bibliometric analysis.

### 3.1 Main information

Table 1 demonstrates the key metrics that emerged from the bibliometric analysis. A total of 13,869 articles were written by 40,656 authors over a 36‐year period with a 9.53% growth rate discerned over the time span, with a generally exponential climb. The period of 36 years considered for the collected articles were segmented into 1985–1997, 1998–2009, and 2010–2021. The greatest growth rate between the three split periods was shown to be between 1985 and 1997 at 12.69%, before declining to 7.19% for 1998–2010, and then increasing in 2010‐2021 to 10.41%.

**Table 1:** Main information about bibliometric analysis, segmented over time periods.

**Data**				
Timespan	1985–2021	1985–1997	1998–2009	2010–2021
Sources	1896	240	549	1590
Documents	13,869	1406	3010	9453
Document average age	10.4	29.5	17.6	5.28
Average citations per doc	15.28	20.5	31.42	9.356
References	200,720	16,077	47,117	157,034
**Document types**				
Original articles	12,254	999	2412	8843
Book chapters	76	0	14	62
Data papers	1	0	0	1
Proceeding papers	353	38	168	147
Meeting abstracts	1185	369	416	400
**Document contents**				
Keyword plus (ID)	10,244	1317	4005	8237
Author's keywords (DE)	13,852	1005	3721	11,548
**Authors**				
Authors	40,656	3134	8756	32,038
Authors of single‐authored docs	577	173	181	248
**Authors collaboration**				
Single‐authored docs	731	218	218	295
Co‐authors per documents	4.9	3.27	4.18	5.37
International co‐authorships %	15.83	3.983	11.43	18.99

### 3.2 Countries, institutions, and departmental productivity

Supporting Information S1: Appendix Figure 2 demonstrates the results of publications in SND between 1985 and 2021 by country of corresponding author. Metadata pertaining to the corresponding author's country was reported in 12734 of 13,869 analyzed articles (Appendices). The most productive countries in terms of scientific output in SND were the United States of America (USA) (*n* = 2610, 18.2%), followed by Brazil (*n* = 971, 6.8%), and Japan (*n* = 778, 5.4%) (Table 2). The USA demonstrated both the highest number of publications which involved collaboration between multiple countries (*n* = 262), as well as no intercountry collaboration (*n* = 2348). However, in terms of the overall proportion of published articles which involved collaborations between multiple countries, Denmark showed the highest, with 59 of 185 studies (32%) being co‐authored by a collaborator in a different geographical location.

**Table 2:** Top 20 most productive countries based on number of published articles.

Ranking by number of articles	Country	Articles, *n* (%)	SCP	MCP (%)
1st	United States of America	2610 (18.2%)	2348	262 (10.0)
2nd	Brazil	971 (6.8%)	827	144 (14.8)
3rd	Japan	778 (5.4%)	690	88 (11.3)
4th	United Kingdom	768 (5.4%)	628	140 (18.2)
5th	India	620 (4.3%	568	52 (8.3)
6th	Australia	554 (3.9%)	437	117 (21.1)
7th	China	502 (3.5%	410	92 (18.3)
8th	Sweden	425 (3.0%)	359	66 (15.5)
9th	Germany	402 (2.8%)	336	66 (16.4)
10th	Canada	401 (2.8%)	339	71 (17.7)
11th	Italy	365 (2.6%)	312	53 (14.5)
12th	Turkey	322 (2.3%)	304	18 (5.6)
13th	Netherlands	266 (1.9%)	204	62 (23.3)
14th	South Korea	234 (1.6%)	200	34 (14.5)
15th	Spain	221 (1.5%)	181	40 (18.1)
16th	Saudi Arabia	218 (1.5%)	153	65 (29.8)
17th	Finland	213 (1.5%)	168	45 (21.1)
18th	Denmark	185 (1.3%)	126	59 (31.9)
19th	Iran	184 (1.3%)	147	37 (20.1)
20th	France	182 (1.3%)	145	37 (20.3)

Abbreviations: MCP, multiple country publications (intercountry collaboration); SCP, single country publications (intra‐country collaboration).

In terms of institutional productivity, The University of Sao Paulo in Brazil published the highest number of articles (*n* = 445), followed by the University of Washington (*n* = 355), the University of North Carolina (*n* = 349), and the University of Adelaide (*n* = 276). A table and plot describing institutional productivity can be found in the appendices (Supporting Information S1: Appendix Tables 6, 7, Appendix Figure A1).

### 3.3 Highly contributive journals, articles, and keywords

Table 3 shows the most productive sources of research in SND in three different time periods. From 1985 to 2009, among the top 10 most productive sources, all sources belong to the field of dentistry. From 2010 to 2021, nine sources were in the field of dentistry, and one in environmental research and public health.

**Table 3:** The top 10 most productive sources based on the number of articles published.

Journal	No. articles	Field
**1985–1997**		
Journal of Dental Research	332 (23.6%)	Dentistry
Community Dentistry and Oral Epidemiology	142 (10.1%)	Dentistry
Journal of The American Dental Association	64 (4.55%)	Dentistry
Journal of Public Health Dentistry	60 (4.27%)	Dentistry
British Dental Journal	47 (3.34%)	Dentistry
Gerodontics	37 (2.63%)	Dentistry
Oral Surgery Oral Medicine Oral Pathology Oral Radiology and Endodontics	34 (2.42%)	Dentistry
Journal of Dentistry For Children	25 (1.78%)	Dentistry
Journal of Periodontology	24 (1.71%)	Dentistry
Acta Odontologica Scandinavica	22 (1.57%)	Dentistry
**1998–2009**		
Journal of Dental Research	331 (11.0%)	Dentistry
Community Dentistry and Oral Epidemiology	115 (3.82%)	Dentistry
Journal of The American Dental Association	109 (3.62%)	Dentistry
British Dental Journal	101 (3.36%)	Dentistry
Journal of Public Health Dentistry	88 (2.92%)	Dentistry
International Dental Journal	86 (2.86%)	Dentistry
Acta Odontologica Scandinavica	73 (2.42%)	Dentistry
Oral Surgery Oral Medicine Oral Pathology Oral Radiology and Endodontology	69 (2.29%)	Dentistry
Journal of Periodontology	67 (2.23%)	Dentistry
Journal of Clinical Periodontology	58 (1.93%)	Dentistry
**2010–2021**		
BMC Oral Health	345 (3.65%)	Dentistry
Gerodontology	294 (3.11%)	Dentistry
Special Care in Dentistry	170 (1.80%)	Dentistry
Community Dentistry and Oral Epidemiology	168 (1.78%)	Dentistry
International Journal of Environmental Research and Public Health	157 (1.66%)	Environmental Research and Public Health
British Dental Journal	147 (1.56%)	Dentistry
Clinical Oral Investigations	137 (1.45%)	Dentistry
Community Dental Health	120 (1.27%)	Dentistry
Medicina Oral Patologia Oral Y Cirugia Bucal	110 (1.16%)	Dentistry
Journal of Public Health Dentistry	107 (1.13%)	Dentistry

Supporting Information S1: Appendix Figures 3–5 illustrated the most cited sources in the references in three different time periods. The “Community Dentistry and Oral Epidemiology” was the most cited source in all three time periods, receiving 12.5%, 54.6%, and 48.5% more citations than the respective second‐most cited sources in the three time periods.

Table 4 and Supporting Information S1: Appendix Table 8 listed the most globally and locally cited articles in each time period, respectively. Of the top 10 most globally cited articles from 1985 to 1997, nine were original studies, and one was a literature review. From 1998 to 2009, six were original articles, and four were reviews (three literature reviews and one systematic review). From 2010 to 2021, three were original articles, and seven were reviews (five literature reviews and two systematic reviews).

**Table 4:** The top 10 articles with the highest global citations.

Document	Year	Local citations	Global citations	Current global citations (April 10, 2024)	LC/GC ratio (%)*	Normalized local citations	Normalized global citations
**1985–1997**							
Beck J, 1996, J Periodontol	1996	2	943	980	0.21	14.52	33.23
Emrich LJ, 1991, J Periodontol	1991	5	390	423	1.28	2.72	12.46
Taylor GW, 1996, J Periodontol	1996	0	342	377	0.00	0.00	12.05
Joshipura KJ, 1996, J Dent Res	1996	1	337	352	0.30	7.26	11.88
Cooper JS, 1995, Int J Radiat Oncol	1995	0	298	329	0.00	0.00	15.51
Urken ML, 1991, Laryngoscope	1991	4	255	260	1.57	2.18	8.14
Mattila KJ, 1995, Clin Infect Dis	1995	1	233	239	0.43	2.77	12.13
Scannapieco FA, 1992, Crit Care Med	1992	0	216	231	0.00	0.00	7.33
Franzen L, 1992, Eur J Cancer	1992	3	194	201	1.55	2.33	6.58
Gritz ER, 1993, Cancer Epidem Biomar	1993	0	193	203	0.00	0.00	5.93
**1998–2009**							
Epstein LJ, 2009, J Clin Sleep Med	2009	0	1771	2110	0.00	0.00	55.22
Marx RE, 2005, J Oral Maxil Surg	2005	45	1056	1124	4.26	19.06	23.64
Bamias A, 2005, J Clin Oncol	2005	45	788	830	5.71	19.06	17.64
Ruggiero SL, 2009, J Oral Maxil Surg	2009	3	713	767	0.42	87.86	22.23
Hoff AO, 2008, J Bone Miner Res	2008	0	455	472	0.00	0.00	14.54
Mavrokokki T, 2007, J Oral Maxil Surg	2007	13	436	450	2.98	16.93	13.64
Bahekar AA, 2007, Am Heart J	2007	2	415	457	0.48	2.61	12.98
Yoneyama T, 2002, J Am Geriatr Soc	2002	20	402	449	4.98	9.92	13.40
Kushida CA, 2006, Sleep	2006	9	374	479	2.41	7.35	11.03
Migliorati CA, 2005, Cancer	2005	35	359	362	9.75	14.83	8.04
**2010–2021**							
Jordan AS, 2014, Lancet	2014	13	607	869	2.14	3.86	42.28
Lockhart PB, 2012, Circulation	2012	66	591	692	11.17	12.82	29.43
Saad F, 2012, Ann Oncol	2012	51	427	499	11.94	9.91	21.26
Chapple ILC, 2013, J Clin Periodontol	2013	0	408	525	0.00	0.00	23.90
Ramar K, 2015, J Clin Sleep Med	2015	23	317	447	7.26	7.15	23.94
Lo JC, 2010, J Oral Maxil Surg	2010	32	291	335	11.00	6.21	13.26
Brown JS, 2010, Lancet Oncol	2010	12	248	322	4.84	2.33	11.30
Epstein JB, 2012, CA‐Cancer J Clin	2012	41	228	303	17.98	7.97	11.35
Petersen PE, 2010, Community Dent Health	2010	85	202	252	42.08	16.50	9.20
Sanz M, 2018, J Clin Periodontol	2018	32	179	348	17.88	20.86	25.70

* LC/GC is the ratio of the number of local citations (citations by articles within the data set) to the number of global citations (citations by all articles). This ratio is indicative of the specificity of the articles in the field of SND.

After merging synonyms and singular/plural forms, and removing irrelevant keywords, the analysis of author's keyword was conducted. Author's keywords are words selected by the authors to represent the central themes of their research.

From 1985 to 1997, “elderly” (*n* = 65), “caries” (*n* = 51), and “epidemiology” (*n* = 38) were the most occurring author's keywords. From 1998 to 2009, they were “elderly” (*n* = 238), “caries” (*n* = 173), and “children” (*n* = 87). From 2010 to 2011, “elderly” (*n* = 883) was the most frequently occurring author's keywords, followed by “caries” (*n* = 682) and “periodontitis” (*n* = 456).

### 3.4 Authors' productivity

Over the period of 1985–2021, the most productive authors were “Locker, D” with 62 published articles, “Tsakos, G” with 56 published articles, and “Berggren, U” with 55 published articles. However, looking at the different time segments (1985–1997, 1998–2009, and 2010–2021), the most productive authors did change. From the first two time segments, “Locker, D “ and “Berggren, U “ both contributed the most articles. In the last time segment, “Tsakos, G” and “Watt, RG” were the most recent highest contributors to the research field. Research findings denoting author productivity can be found in the Supporting Information S1: Appendix Tables 1–5.

The authors with the highest indices are “Locker, D” with an *h*‐index of 32 and *g*‐index of 51, a total citation of 2700, “Tsakos, G” with an *h*‐index of 24, *g*‐index of 43, a total citation of 1955, and “Berggren, U” with an *h*‐index of 24, *g*‐index of 38, and total citation of 1586.

## 4 DISCUSSION

This comprehensive bibliometric analysis has provided perspective of the scope of research in SND over the past 36 years, since the conception of this specialty. To the best of our knowledge, this analysis quantifies the progress and the current breadth of existing research in SND and is the first of its kind.

Research activity on SND escalated over the period examined, a testament to gradual recognition, interest and growth in the speciality, as well as an attempt to define SND. In 1981, under the auspices of the American Dental Association (ADA), several journals were consolidated to form “Special Care in Dentistry” (Ettinger et al., 2004). Despite capturing metadata in this study proceeding the year 1985, preliminary recognition of special care dentistry through dedicated journals could explain the greatest growth rate in 1985–1997. Additionally, establishing the definition of Special Care Dentistry and the synonymising of “special care” and “special needs” cornerstones the rise in publications during the timespan (Ettinger, 2000). By 1994, there was unanimity globally on the need for a career structure for persons working in SND (Dental Board of Australia ‐ Registration Standards Internet, 2023). The recognition by organized dentistry and academia, and dedicated programs established in the USA, Great Britain, Australia, New Zealand, and several nations in Europe and South America further accounts for the growth in publications during the time of 1998–2010 (Derbi & Borromeo, 2016). 2008 onward saw tremendous exponential growth in published articles, following the adoption of the SND definition and the scope of practice in the speciality concerns (Shnider, 2008). Furthermore, affiliate professional organizations have advocated to refine the clinical care provisions for patients with special needs, as well as education, professional development, policies, and research over the past decade; akin to the exponential document growth of the 2010–2021 period (Chalrners, 2001). However despite such revelations, the transition of research and knowledge into clinical applications appears inadequate with issues of inaccessibility, discrimination, and discrepancies between theory and practice still present today (Scambler & Curtis, 2019).

In terms of countries' research productivity on SND, the USA leads the field among the majority of countries in the world that have contributed. This is consistent with the earliest recognition of special care dentistry, which was termed in collaboration with several journals originally based in the USA (Ettinger et al., 2004). Interestingly, the majority of studies originating from the USA are based only in American institutions, with low instances of collaboration with authors based in other countries. It is worth noting that despite the USA having shown the greatest research contribution, it does not recognize SND as a specialty. There is currently a lack of formal training for dentists in both undergraduate and postgraduate dental curricula regarding the provision of dental services to special needs individuals. Furthermore, there is no adequate career pathway or recognition for those working with patients with special needs, and therefore such individuals are not considered specialists (Ettinger et al., 2004). There have been fellowships for Geriatric Dentistry or Dentistry for those with disabilities; however, these programs aren't officially recognized by the ADA. Brazil has demonstrated significant research contributions and was the first to recognize Special Care Dentistry as a specialty as of 2001. This decision was driven by the increase in the number of people with disabilities and their demands for dental care. Reportedly, many dental schools in Brazil do not provide adequate training in SND for undergraduate students, resulting in a shortage of dental professionals equipped to treat these patients and increased demand for specialists (Mugayar et al., 2007). In contrast, there has been a strong research drive emerging from Japan in the field of Geriatric Dentistry. Improvements in social environments have resulted in the highest life‐expectancy and fastest aging society worldwide (Kitagawa et al., 2011). As populations around the world continue to age due to advances in medicine and the social environment, greater demand is expected worldwide for those specialized in providing services to older patients. Similarly to the USA, Japan does not recognize SND as a formal specialty. Countries which recognize SND as a specialty include Australia, New Zealand, Malaysia, Brazil, and the Great Britain (Ettinger et al., 2004).

With advancements in technology allowing easier dissemination of scientific information, there are more studies being published which involve collaboration between authors from different geographic locations. However, publications involving multiple country collaborations are still relatively few. This can be explained by the difference in the recognition of SND in different countries, the different prevalences of special needs of each country's population, and differing modalities of treatment and care. There is yet to be one single universally accepted definition for SND. With regard to countries which have formally recognized the specialty, the specialist workforce still remains few with significant demand for services (Ettinger et al., 2004; Mugayar et al., 2007). Clinicians are often required to focus on clinical practice to address population needs, which may explain the limited amount of studies emerging from some of these countries. Further increases in global scientific productivity and multi‐national collaborations would have a stronger impact in advancing the field of knowledge, as it can effectively boost academic recruitment, as well as promotion and funding, benefitting all relevant stakeholders.

Interestingly, the institutional affiliations of authors involved in research output pertaining to SND are based largely in the departments or faculties of dentistry, and the majority of productive journals were dental journals. Given the bidirectional relationship between dentistry and various special and social conditions under special needs (Lim et al., 2020), SND requires multidisciplinary contribution. Our results indicate input from other disciplines' into SND is currently limited, and assimilation of a social model approach into the current research may be pragmatic (Scambler & Curtis, 2019).

From the first to the third timespan, the percentage of review articles in the top 20 most globally cited articles increased from 10% to 33% and lastly to 50%. In the first timespan, the total number of SND articles was only 1406, which is a small foundation to produce reviews from. In the following two timespans, the growth in the knowledge base allowed the production of more, higher quality reviews with in‐built analysis of multiple original articles and bias analysis. Overall, the research of SND evolved from mainly laying the foundation with original articles in the earlier years to producing more review articles in the later years, indicating a maturing research scene. The analyses of author's keywords indicated that “elderly” and “caries” reached the highest frequency in all three time spans. This mirrors aging and caries being the most prevalent special needs and oral health disease (Benjamin, 2010). Additionally, there has been an increasing trend of tooth retention with age (Atanda et al., 2022). Together with the close relationship between oral health and aging, and its accompanying loss of functional independence, chronic disease, and polypharmacy, this explains aging as a persisting topic of interest. From 1985 to 1997, “epidemiology” was the third most frequently occurring keyword, indicating that incidence and distribution studies were a large focus in these years (Jindal, 2013). In the next two timespans, “epidemiology” no longer appeared as frequently, reflecting maturation of the SND research scene. “Children” as the second‐most prevalent special needs (excluding healthy children” and “periodontitis” as the next prevailing oral health disease placed them as the second and third most frequently occurring keywords from 1998 to 2009 and 2010 to 2011. The emergence of periodontitis in the more recent years could be explained by the progressive understanding of the association between periodontal health and other chronic diseases such as diabetes mellitus (Chee et al., 2013), rheumatoid arthritis (Bartold et al., 2005), dementia (Noble et al., 2009), and cardiovascular diseases (Beck & Offenbacher, 2001). Overall, the result is unsurprising and implies that more research input into the less prevalent conditions would be beneficial.

Given the inherent complexities of bibliometric studies, it is expected that there will be occurrences of false‐positives and false‐negatives, as achieving a flawless search strategy is highly challenging. Furthermore, the search is intended to be run only on the Web of Science database due to the availability of metadata, and therefore is not exhaustive. Only including articles in the English language may have also underestimated the productivity of countries publishing in other languages. Furthermore, exclusive screening by title may have resulted in the inclusion of irrelevant articles and exclusion of relevant articles. Our analyses represent an objective and quantitative view on the field; however, this does not take into account the quality of the individual studies nor any influence on clinical practice. Nonetheless, the study could pioneer an approach to large‐scale mature literature analysis of other dental disciplines or even within particular populations of SND. This bibliometric analysis established a methodology for replication whereby an automated, flexible, and rapidly updating workflow can be applied easily to output valuable information on trends, correlations, keywords, and productivity.

## AUTHOR CONTRIBUTIONS

**Nigashiny Senthilvadevel** and **Jimmy Ky**: Contributed to acquisition, analysis, and interpretation, drafted the manuscript, critically revised manuscript, and gave final approval. **Matthew Ng, Tong Zhao, Massimo Aria, and Luca D'Aniello**: Contributed to acquisition, analysis, and interpretation, drafted the manuscript and gave final approval. **Mathew A. W. Lim**: Contributed to acquisition, critically revised the manuscript and gave final approval. **Federica Canfora, Giulio Fortuna, Michael McCullough**, and **Tami Yap**: Contributed to interpretation, critically revised manuscript, and gave final approval. **Rita Paolini**, and **Antonio Celentano**: Contributed to conception and design, contributed to acquisition, analysis, and interpretation, drafted the manuscript, critically revised manuscript, and gave final approval.

## CONFLICT OF INTEREST STATEMENT

The authors have no conflict of interest to declare.

## ETHICS STATEMENT

The authors have nothing to report.

## Supporting information

Supporting information.

## Data Availability

The data that support the findings of this study are available from the corresponding author upon reasonable request.
