# Supplementary material for: Evaluating global research trends in special needs dentistry: A systematic bibliometrix analysis
Source: Clin Exp Dent Res. 2024 Jun 16;10(3):e896. doi: 10.1002/cre2.896 (PMC11180849; doi:10.1002/cre2.896)
Supplement: Supplementary file 1 — Supporting information. [file CRE2-10-e896-s001.docx]

**Appendix**

### **Article Title:** Evaluating global research trends in Special Needs Dentistry: A systematic bibliometrix analysis

**Appendix Figure 1.** PRISMA flow diagram of process of identification and screening of articles to be included in bibliometrix analysis


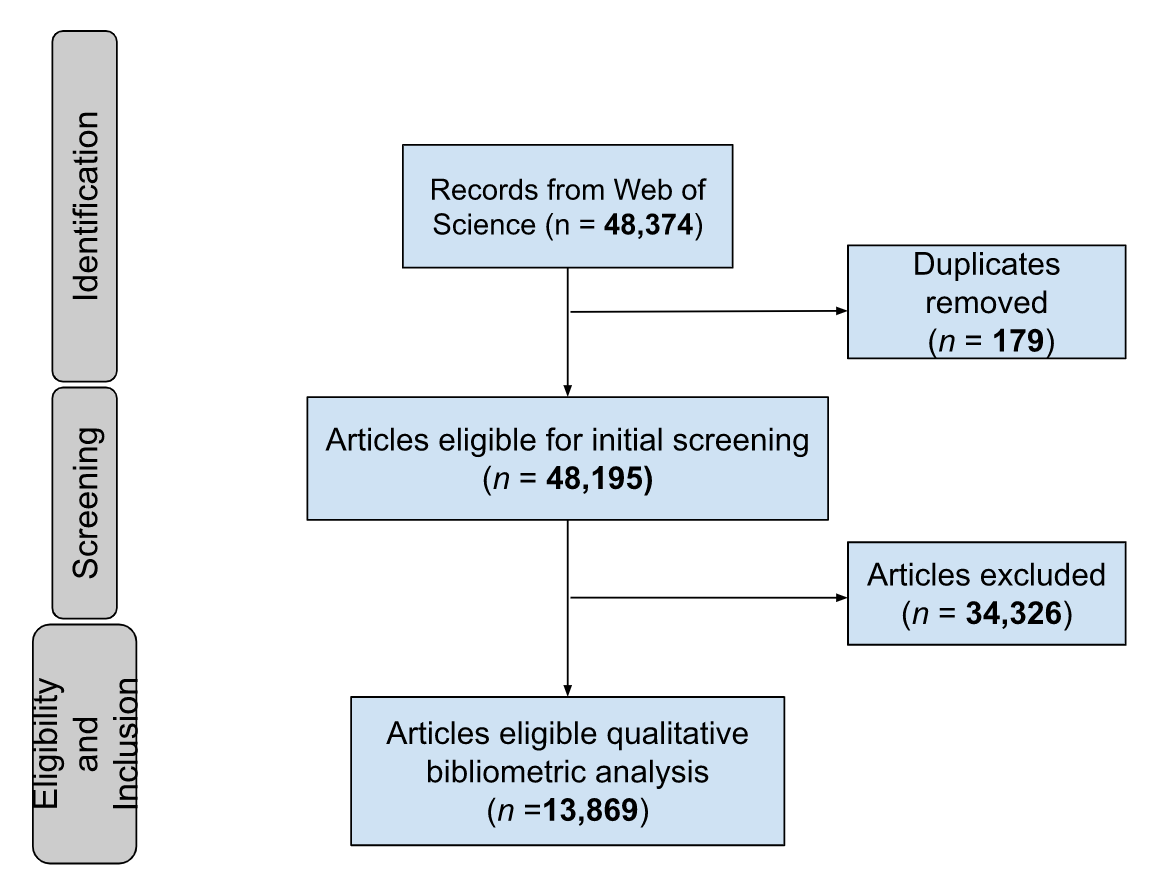


**Appendix Figure 2.** Country of origin of published articles regarding dentistry pertinent to special needs populations. Gray indicates a total absence of published articles originating from that country.


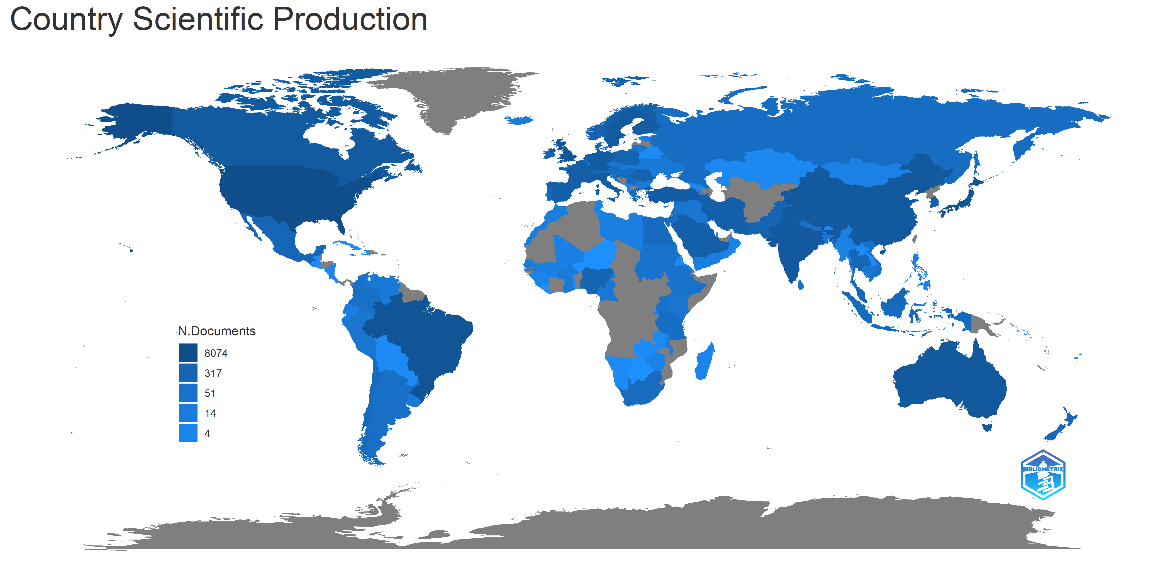


**Appendix Figure 3.** The most locally cited sources from 1985 to 1997


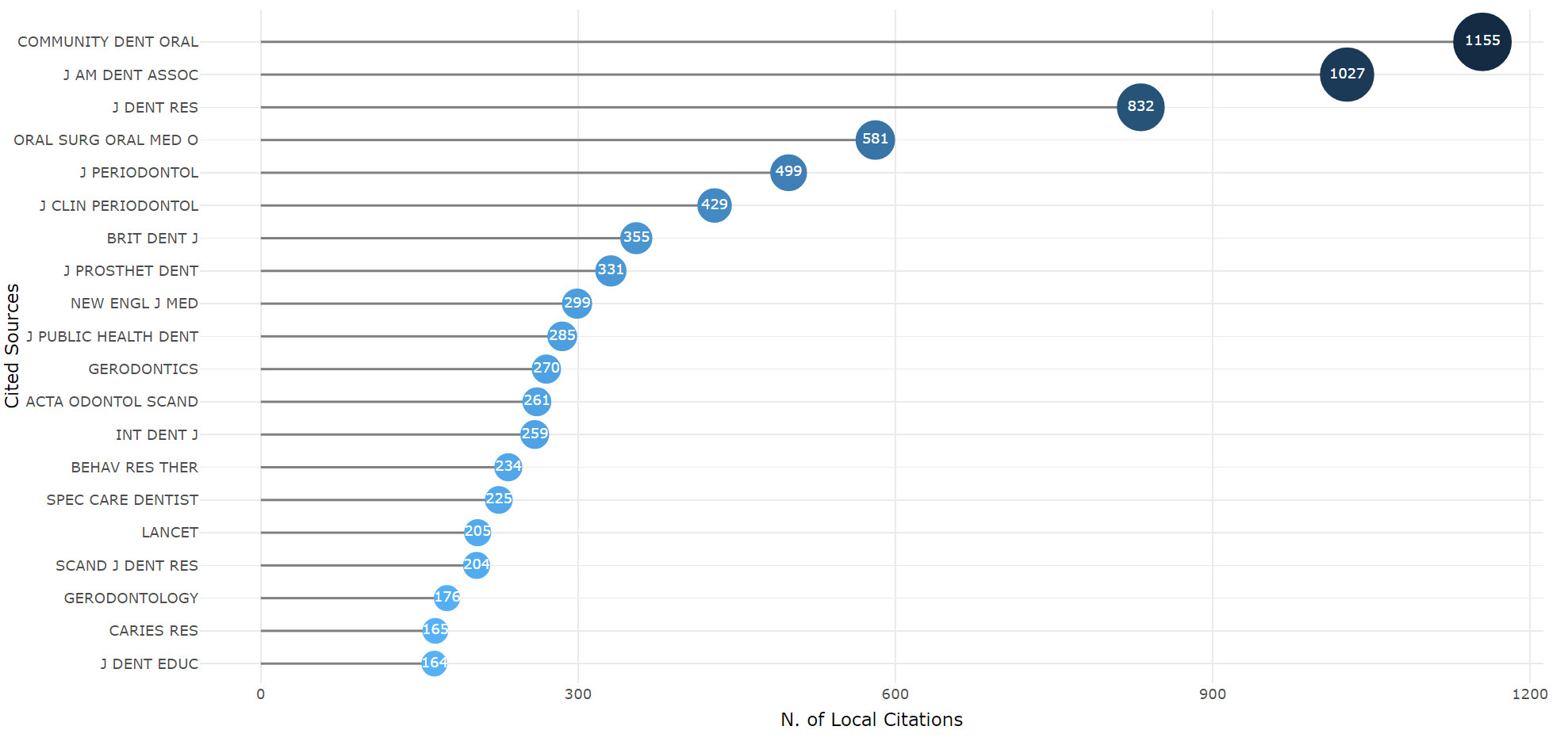


**Appendix Figure 4.** The most locally cited sources from 1998 to 2009


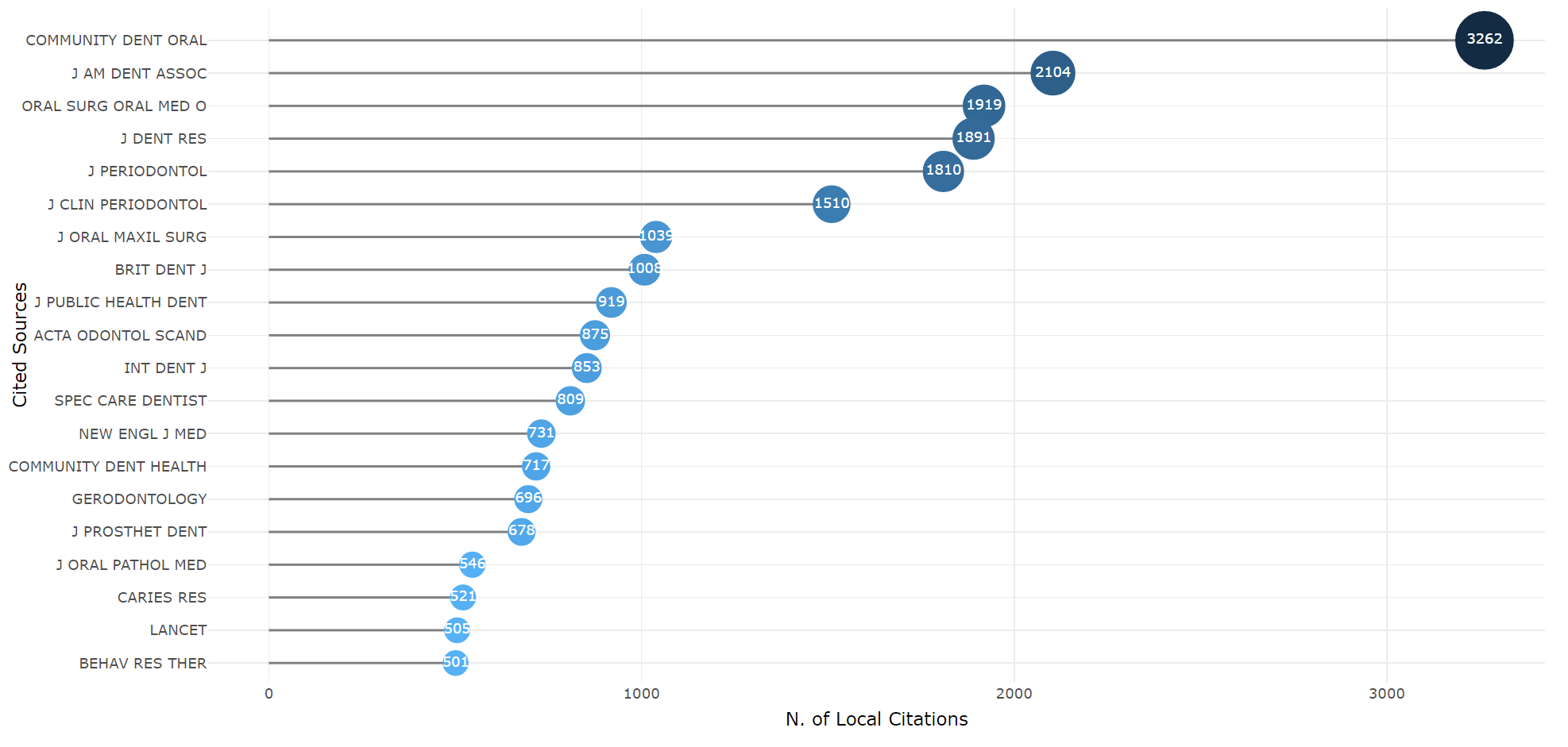


**Appendix Figure 5.** The most locally cited sources from 2010 to 2021


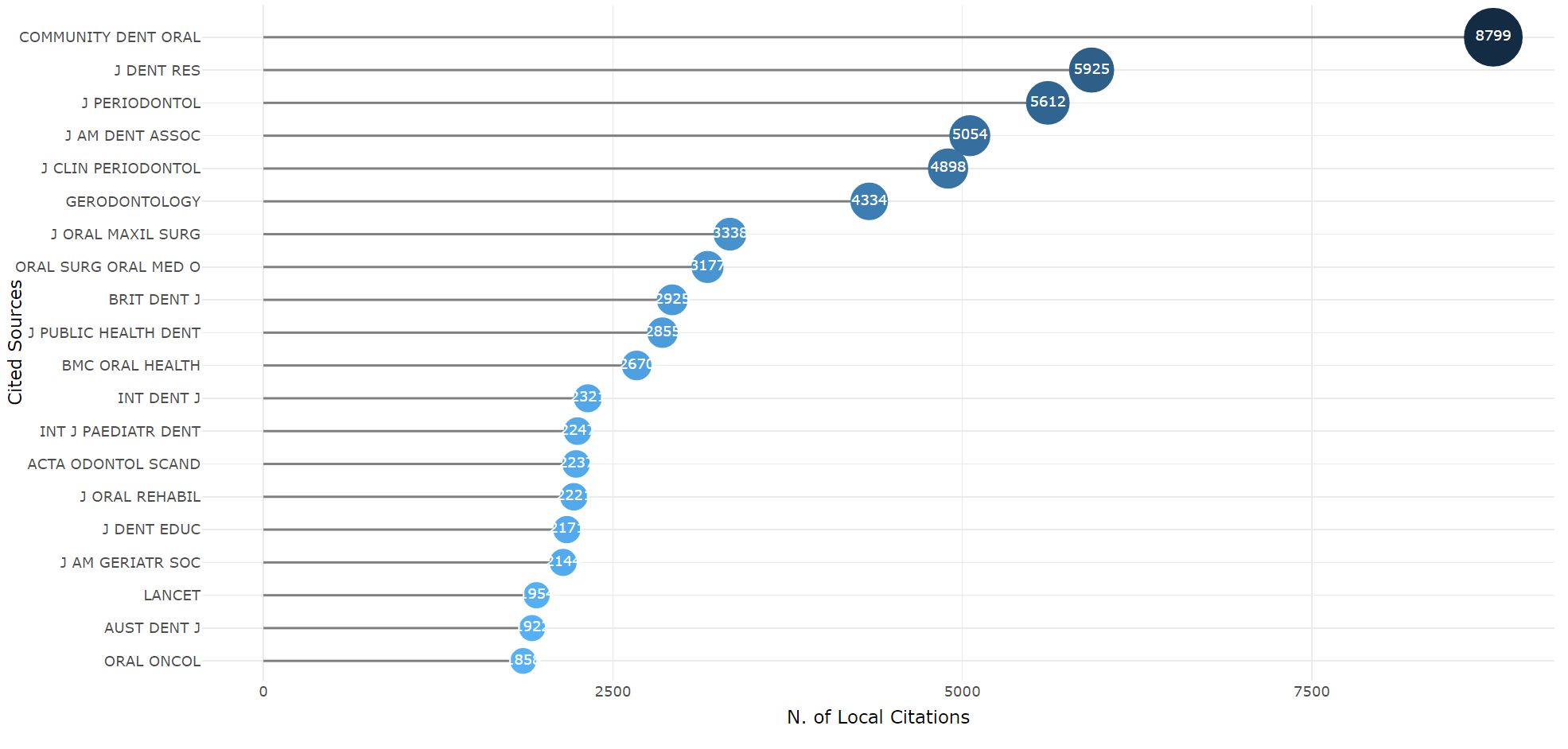


**Search Strategy - Web of Science**

| *“intellectual* disab*” or “physical* disab*” or syndromic or neuro* or cognitive or “mental disorder*” or psychiatric or “mental illness*” or “special need*” or elderly or geriatric* or “older adult*” or “down syndrome*” or epilep* or autis* or asperger* or “pervasive developmental disorder*” or “child* disintegrative” or “prader-willi syndrome*” or “angelman* syndrome*or fragile x” or “fetal alcohol” or Parkinson* or stroke* or “multiple sclerosis*” or “motor neurone*” or “huntington* disease” or “spinocerebellar ataxia” or “acquired brain injur*” or “cerebral palsy” or quadriplegi* or schizophren* or bipolar or depress* or anxi* or phobia or fear or “obsessive convulsive” or “conduct disorder*” or “post-traumatic stress*” or “posttraumatic stress*” or “personality disorder*” or substance* or addict* or “behavioural disorder*” or “sensory deficit*” or “sensory disorder*” or “sensory impair*” or “hearing impair*” or “phenylketonuria” or “cystic fibrosis” or “osteogenesis imperfecta” or transplant or cancer or oncolog* or radiotherapy or chemotherapy or immunotherapy or immunodeficiency or diabet* or bleed* or haemophil* or “von Willebrand” or HIV or AIDS or socio-economic or socioeconomic or poverty or “low income” or minority or disadvantaged or immigrant or rural or prison* or jail or incarcera* or crim* or homeless* or “child* with special needs” or “minor* with special needs” or “special needs child*” or “special needs minor*” or insomnia* or sleep or speech or mute or communic* or “frail elder*” or dementia* or Alzheimer* or domiciliary or palliative or dysphagia or “age* care” or “medical* complicat*”* |
| --- |
| *AND* |
| *dentist* or dental or “oral health*” or “special care dentist*” or “special needs dentist*”* |

**Appendix Table 1.** Top 10 most productive authors based on number of published articles from 1985-2021

| **Authors** | **Articles** | **Articles Fractionalized** |
| --- | --- | --- |
| LOCKER D | 62 | 28.17 |
| TSAKOS G | 56 | 10.25 |
| BERGGREN U | 55 | 17.92 |
| MIYAZAKI H | 53 | 9.71 |
| HAKEBERG M | 49 | 14.08 |
| WU B | 48 | 12.09 |
| ASTROM AN | 47 | 11.25 |
| WATT RG | 47 | 7.96 |
| YOSHIHARA A | 44 | 8.42 |
| MEURMAN JH | 43 | 7.83 |

**Appendix Table 2.** Top 10 most productive authors based on number of published articles from 1985-1997

| **Authors** | **Articles** | **Articles Fractionalized** |
| --- | --- | --- |
| LOCKER D | 30 | 14.90 |
| BERGGREN U | 24 | 9.65 |
| BECK JD | 21 | 6.52 |
| MILGROM P | 15 | 4.30 |
| SCULLY C | 15 | 3.90 |
| HUNT RJ | 14 | 4.70 |
| SLADE GD | 14 | 5.28 |
| CARLSSON SG | 13 | 3.73 |
| KUNZEL C | 13 | 6.00 |
| SADOWSKY D | 13 | 6.00 |

**Appendix Table 3.** Top 10 most productive authors based on number of published articles from 1989-2009

| **Authors** | **Articles** | **Articles Fractionalized** |
| --- | --- | --- |
| LOCKER D | 32 | 13.27 |
| BERGGREN U | 28 | 7.65 |
| DE JONGH A | 19 | 5.94 |
| MIYAZAKI H | 19 | 3.88 |
| FRIEDLANDER AH | 17 | 5.78 |
| FISKE J | 16 | 6.60 |
| MEURMAN JH | 16 | 3.32 |
| EPSTEIN JB | 15 | 4.83 |
| HAKEBERG M | 15 | 3.81 |
| HANADA N | 15 | 3.53 |

**Appendix Table 4.** Top 10 most productive authors based on number of published articles from 2010-2021

| **Authors** | **Articles** | **Articles Fractionalized** |
| --- | --- | --- |
| TSAKOS G | 48 | 8.53 |
| WATT RG | 44 | 6.96 |
| WU B | 41 | 9.50 |
| AIDA J | 37 | 5.06 |
| FOLAYAN MO | 35 | 7.17 |
| ASTROM AN | 33 | 7.59 |
| JAMIESON LM | 32 | 7.14 |
| MIYAZAKI H | 32 | 5.46 |
| YOSHIHARA A | 32 | 5.98 |
| HIRANO H | 30 | 3.76 |

**Appendix Table 5.** Top 10 most influential authors based on h-index, g-index, and total citations

| **Authors** | **h-index** | **g-index** | **Total citations** |
| --- | --- | --- | --- |
| LOCKER D | 32 | 51 | 2700 |
| BERGGREN U | 24 | 38 | 1586 |
| TSAKOS G | 24 | 43 | 1955 |
| BECK JD | 23 | 39 | 1525 |
| HAKEBERG M | 22 | 33 | 1196 |
| SHEIHAM A | 21 | 29 | 1505 |
| MIYAZAKI H | 20 | 34 | 1271 |
| THOMSON WM | 20 | 30 | 931 |
| GILBERT GH | 19 | 28 | 875 |
| MEURMAN JH | 19 | 31 | 1042 |

**Appendix Table 6.** Scientific Production per country

​​

| **Country** | **Articles** | **SCP** | **MCP** | **MCP Ratio** |
| --- | --- | --- | --- | --- |
| USA | 2610 | 2348 | 262 | 0.1 |
| BRAZIL | 971 | 827 | 144 | 0.148 |
| JAPAN | 778 | 690 | 88 | 0.113 |
| UNITED KINGDOM | 768 | 628 | 140 | 0.182 |
| INDIA | 620 | 568 | 52 | 0.084 |
| AUSTRALIA | 554 | 437 | 117 | 0.211 |
| CHINA | 502 | 410 | 92 | 0.183 |
| SWEDEN | 425 | 359 | 66 | 0.155 |
| GERMANY | 402 | 336 | 66 | 0.164 |
| CANADA | 401 | 330 | 71 | 0.177 |
| ITALY | 365 | 312 | 53 | 0.145 |
| TURKEY | 322 | 304 | 18 | 0.056 |
| NETHERLANDS | 266 | 204 | 62 | 0.233 |
| KOREA | 234 | 200 | 34 | 0.145 |
| SPAIN | 221 | 181 | 40 | 0.181 |
| SAUDI ARABIA | 218 | 153 | 65 | 0.298 |
| FINLAND | 213 | 168 | 45 | 0.211 |
| DENMARK | 185 | 126 | 59 | 0.319 |
| IRAN | 184 | 147 | 37 | 0.201 |
| FRANCE | 182 | 145 | 37 | 0.203 |
| NORWAY | 169 | 88 | 81 | 0.479 |
| ISRAEL | 139 | 122 | 17 | 0.122 |
| ROMANIA | 126 | 119 | 7 | 0.056 |
| POLAND | 122 | 115 | 7 | 0.057 |
| NIGERIA | 97 | 75 | 22 | 0.227 |
| SWITZERLAND | 97 | 60 | 37 | 0.381 |
| PAKISTAN | 94 | 80 | 14 | 0.149 |
| IRELAND | 86 | 57 | 29 | 0.337 |
| MEXICO | 84 | 60 | 24 | 0.286 |
| MALAYSIA | 82 | 58 | 24 | 0.293 |
| BELGIUM | 73 | 48 | 25 | 0.342 |
| THAILAND | 73 | 59 | 14 | 0.192 |
| INDONESIA | 63 | 50 | 13 | 0.206 |
| CROATIA | 62 | 55 | 7 | 0.113 |
| NEW ZEALAND | 58 | 41 | 17 | 0.293 |
| SERBIA | 56 | 55 | 1 | 0.018 |
| GREECE | 55 | 31 | 24 | 0.436 |
| SOUTH AFRICA | 52 | 37 | 15 | 0.288 |
| PORTUGAL | 43 | 31 | 12 | 0.279 |
| AUSTRIA | 42 | 30 | 12 | 0.286 |
| EGYPT | 37 | 28 | 9 | 0.243 |
| CHILE | 36 | 19 | 17 | 0.472 |
| JORDAN | 35 | 16 | 19 | 0.543 |
| RUSSIA | 35 | 31 | 4 | 0.114 |
| HUNGARY | 28 | 24 | 4 | 0.143 |
| SINGAPORE | 28 | 22 | 6 | 0.214 |
| TANZANIA | 26 | 18 | 8 | 0.308 |
| COLOMBIA | 23 | 14 | 9 | 0.391 |
| KUWAIT | 22 | 16 | 6 | 0.273 |
| U ARAB EMIRATES | 21 | 16 | 5 | 0.238 |
| SRI LANKA | 17 | 14 | 3 | 0.176 |
| ETHIOPIA | 16 | 12 | 4 | 0.25 |
| NEPAL | 16 | 14 | 2 | 0.125 |
| SUDAN | 16 | 6 | 10 | 0.625 |
| ARGENTINA | 15 | 10 | 5 | 0.333 |
| IRAQ | 15 | 14 | 1 | 0.067 |
| UGANDA | 14 | 10 | 4 | 0.286 |
| LITHUANIA | 13 | 9 | 4 | 0.308 |
| SYRIA | 13 | 12 | 1 | 0.077 |
| BENIN | 12 | 11 | 1 | 0.083 |
| BULGARIA | 12 | 12 | 0 | 0 |
| KENYA | 11 | 9 | 2 | 0.182 |
| UKRAINE | 11 | 11 | 0 | 0 |
| LEBANON | 10 | 9 | 1 | 0.1 |
| HONG KONG | 9 | 8 | 1 | 0.111 |
| VIETNAM | 8 | 5 | 3 | 0.375 |
| BOSNIA | 7 | 4 | 3 | 0.429 |
| CZECH REPUBLIC | 7 | 6 | 1 | 0.143 |
| VENEZUELA | 7 | 6 | 1 | 0.143 |
| CAMEROON | 6 | 2 | 4 | 0.667 |
| PERU | 6 | 3 | 3 | 0.5 |
| SLOVENIA | 6 | 4 | 2 | 0.333 |
| BANGLADESH | 5 | 1 | 4 | 0.8 |
| GHANA | 5 | 3 | 2 | 0.4 |
| MOROCCO | 5 | 5 | 0 | 0 |
| URUGUAY | 5 | 4 | 1 | 0.2 |
| CAMBODIA | 4 | 1 | 3 | 0.75 |
| ICELAND | 4 | 3 | 1 | 0.25 |
| JAMAICA | 4 | 3 | 1 | 0.25 |
| KOSOVO | 4 | 4 | 0 | 0 |
| MONTENEGRO | 4 | 0 | 4 | 1 |
| QATAR | 4 | 2 | 2 | 0.5 |
| SENEGAL | 4 | 4 | 0 | 0 |
| SLOVAKIA | 4 | 3 | 1 | 0.25 |
| ZIMBABWE | 4 | 4 | 0 | 0 |
| BURKINA FASO | 3 | 1 | 2 | 0.667 |
| ESTONIA | 3 | 0 | 3 | 1 |
| GEORGIA | 3 | 2 | 1 | 0.333 |
| LIBYA | 3 | 3 | 0 | 0 |
| MALTA | 3 | 2 | 1 | 0.333 |
| ARMENIA | 2 | 1 | 1 | 0.5 |
| CONGO | 2 | 1 | 1 | 0.5 |
| ECUADOR | 2 | 1 | 1 | 0.5 |
| MACEDONIA | 2 | 1 | 1 | 0.5 |
| OMAN | 2 | 1 | 1 | 0.5 |
| PARAGUAY | 2 | 0 | 2 | 1 |
| TUNISIA | 2 | 2 | 0 | 0 |
| BHUTAN | 1 | 0 | 1 | 1 |
| BOTSWANA | 1 | 1 | 0 | 0 |
| BRUNEI | 1 | 0 | 1 | 1 |
| COSTA RICA | 1 | 0 | 1 | 1 |
| ERITREA | 1 | 1 | 0 | 0 |
| GUINEA | 1 | 1 | 0 | 0 |
| LESOTHO | 1 | 0 | 1 | 1 |
| MALAWI | 1 | 1 | 0 | 0 |
| MALI | 1 | 1 | 0 | 0 |
| MOLDOVA | 1 | 1 | 0 | 0 |
| MONGOLIA | 1 | 0 | 1 | 1 |
| MYANMAR | 1 | 1 | 0 | 0 |
| PANAMA | 1 | 1 | 0 | 0 |
| PHILIPPINES | 1 | 0 | 1 | 1 |
| RWANDA | 1 | 1 | 0 | 0 |
| YEMEN | 1 | 1 | 0 | 0 |
| ZAMBIA | 1 | 0 | 1 | 1 |
| UNREPORTED/MISSING | 1568 | 1503 | 65 | 0.041 |

Abbreviations: MCP: multiple country publications (inter-country collaboration); SCP: single country publications (intra-country collaboration).

**Appendix Table 7.** Top 20 most productive affiliations (Affiliation Name Disambiguation Disabled)

| **Affiliations** | **Articles** |
| --- | --- |
| UNIV HONG KONG FAC DENT HONG KONG HONG KONG PEOPLES R CHINA | 53 |
| UCL DEPT EPIDEMIOL & PUBL HLTH LONDON ENGLAND | 37 |
| VRIJE UNIV AMSTERDAM AMSTERDAM NETHERLANDS | 33 |
| UNIV N CAROLINA SCH DENT DEPT DENT ECOL CHAPEL HILL NC 27599 USA | 32 |
| UNIV CALIF LOS ANGELES SCH DENT LOS ANGELES CA 90024 USA | 29 |
| UNIV MICHIGAN SCH DENT DEPT PERIODONT & ORAL MED ANN ARBOR MI 48109 USA | 28 |
| UNIV ADELAIDE SCH DENT AUSTRALIAN RES CTR POPULAT ORAL HLTH ADELAIDE SA 5005 AUSTRALIA | 26 |
| OULU UNIV HOSP ORAL & MAXILLOFACIAL DEPT OULU FINLAND | 25 |
| UNIV ADELAIDE AUSTRALIAN RES CTR POPULAT ORAL HLTH ADELAIDE SA 5005 AUSTRALIA | 25 |
| MCGILL UNIV FAC DENT MONTREAL PQ CANADA | 24 |
| UNIV MELBOURNE MELBOURNE DENT SCH MELBOURNE VIC AUSTRALIA | 22 |
| UNIV TORONTO FAC DENT TORONTO ON CANADA | 22 |
| UNIV ADELAIDE SCH DENT AUSTRALIAN RES CTR POPULAT ORAL HLTH ADELAIDE SA AUSTRALIA | 21 |
| UNIV MICHIGAN SCH DENT ANN ARBOR MI 48109 USA | 21 |
| UNIV WASHINGTON SEATTLE WA 98195 USA | 20 |
| UCL DEPT EPIDEMIOL & PUBL HLTH LONDON WC1E 6BT ENGLAND | 19 |
| UNIV EASTERN FINLAND INST DENT KUOPIO FINLAND | 18 |
| UNIV WASHINGTON SCH DENT SEATTLE WA 98195 USA | 18 |
| KAROLINSKA INST DEPT DENT MED STOCKHOLM SWEDEN | 17 |
| NYU COLL DENT DEPT EPIDEMIOL & HLTH PROMOT NEW YORK NY USA | 17 |
| NYU COLL DENT NEW YORK NY USA | 17 |
| KINGS COLL LONDON FAC DENT ORAL & CRANIOFACIAL SCI LONDON ENGLAND | 16 |
| UNIV BRITISH COLUMBIA FAC DENT VANCOUVER BC CANADA | 16 |
| UNIV HELSINKI INST DENT FIN-00014 HELSINKI FINLAND | 16 |
| UNIV HONG KONG FAC DENT HONG KONG PEOPLES R CHINA | 16 |

**Appendix Table 8.** The top ten articles with the highest local citations

| **Document** | **Year** | **Local Citations** | **Global Citations** | **LC/GC Ratio (%)** | **Normalized Local Citations** | **Normalized Global Citations** |
| --- | --- | --- | --- | --- | --- | --- |
| **1985-1997** | | | | | | |
| GERBERT B, 1987, J AM DENT ASSOC | 1987 | 29 | 92 | 31.52 | 18.50 | 10.99 |
| MOORE R, 1991, BEHAV RES THER | 1991 | 21 | 104 | 20.19 | 11.44 | 3.32 |
| BERGGREN U, 1985, COMMUNITY DENT ORAL | 1985 | 18 | 59 | 30.51 | 6.60 | 4.03 |
| BRAUER L, 1986, COMMUNITY DENT ORAL | 1986 | 14 | 30 | 46.67 | 12.51 | 4.32 |
| KLEIN RS, 1988, NEW ENGL J MED | 1988 | 14 | 172 | 8.14 | 7.46 | 13.92 |
| GERBERT B, 1988, J PUBLIC HEALTH DENT | 1988 | 14 | 35 | 40.00 | 7.46 | 2.83 |
| GREENSPAN JS, 1992, ORAL SURG ORAL MED O | 1992 | 12 | 145 | 8.28 | 9.33 | 4.92 |
| GERBERT B, 1988, J AM DENT ASSOC | 1988 | 11 | 38 | 28.95 | 5.86 | 3.08 |
| MOORE R, 1991, COMMUNITY DENT ORAL | 1991 | 11 | 61 | 18.03 | 5.99 | 1.95 |
| LOCKER D, 1993, COMMUNITY DENT ORAL | 1993 | 11 | 50 | 22.00 | 8.27 | 1.54 |
| **1998-2009** | | | | | | |
| BAMIAS A, 2005, J CLIN ONCOL | 2005 | 45 | 788 | 5.71 | 19.06 | 17.64 |
| MARX RE, 2005, J ORAL MAXIL SURG | 2005 | 45 | 1056 | 4.26 | 19.06 | 23.64 |
| MIGLIORATI CA, 2005, CANCER | 2005 | 35 | 359 | 9.75 | 14.83 | 8.04 |
| WU TJ, 2000, ARCH INTERN MED | 2000 | 25 | 328 | 7.62 | 9.23 | 11.23 |
| JOSHIPURA KJ, 2003, STROKE | 2003 | 23 | 248 | 9.27 | 13.63 | 9.08 |
| HELLSTEIN JW, 2005, J ORAL MAXIL SURG | 2005 | 23 | 183 | 12.57 | 9.74 | 4.10 |
| MIGLIORATI CA, 2005, J AM DENT ASSOC | 2005 | 23 | 281 | 8.19 | 9.74 | 6.29 |
| SHEIHAM A, 2001, COMMUNITY DENT ORAL | 2001 | 22 | 167 | 13.17 | 8.72 | 5.32 |
| DEVANI P, 1998, BRIT J ORAL MAX SURG | 1998 | 21 | 136 | 15.44 | 9.35 | 6.47 |
| LOCKER D, 1999, J DENT RES | 1999 | 20 | 154 | 12.99 | 5.45 | 4.74 |
| **2010-2021** | | | | | | |
| PETERSEN PE, 2010, COMMUNITY DENT HLTH | 2010 | 85 | 202 | 42.08 | 16.50 | 9.20 |
| JABER MA, 2011, J APPL ORAL SCI | 2011 | 68 | 107 | 63.55 | 12.50 | 5.08 |
| GRIFFIN SO, 2012, AM J PUBLIC HEALTH | 2012 | 67 | 159 | 42.14 | 13.02 | 7.92 |
| LOCKHART PB, 2012, CIRCULATION | 2012 | 66 | 591 | 11.17 | 12.82 | 29.43 |
| THOMSON WM, 2014, GERODONTOLOGY | 2014 | 54 | 106 | 50.94 | 16.04 | 7.38 |
| NELSON LP, 2011, PEDIATR DENT | 2011 | 53 | 97 | 54.64 | 9.74 | 4.61 |
| KAYE EK, 2010, J AM GERIATR SOC | 2010 | 51 | 146 | 34.93 | 9.90 | 6.65 |
| SAAD F, 2012, ANN ONCOL | 2012 | 51 | 427 | 11.94 | 9.91 | 21.26 |
| GIL-MONTOYA JA, 2015, CLIN INTERV AGING | 2015 | 50 | 155 | 32.26 | 15.54 | 11.70 |
| LISTL S, 2011, J DENT RES | 2011 | 46 | 101 | 45.54 | 8.46 | 4.80 |

**Appendix Figure A1.** Most relevant affiliations (Top 20) and affiliations production over time (Top 5) (Affiliation Name Disambiguation Enabled)


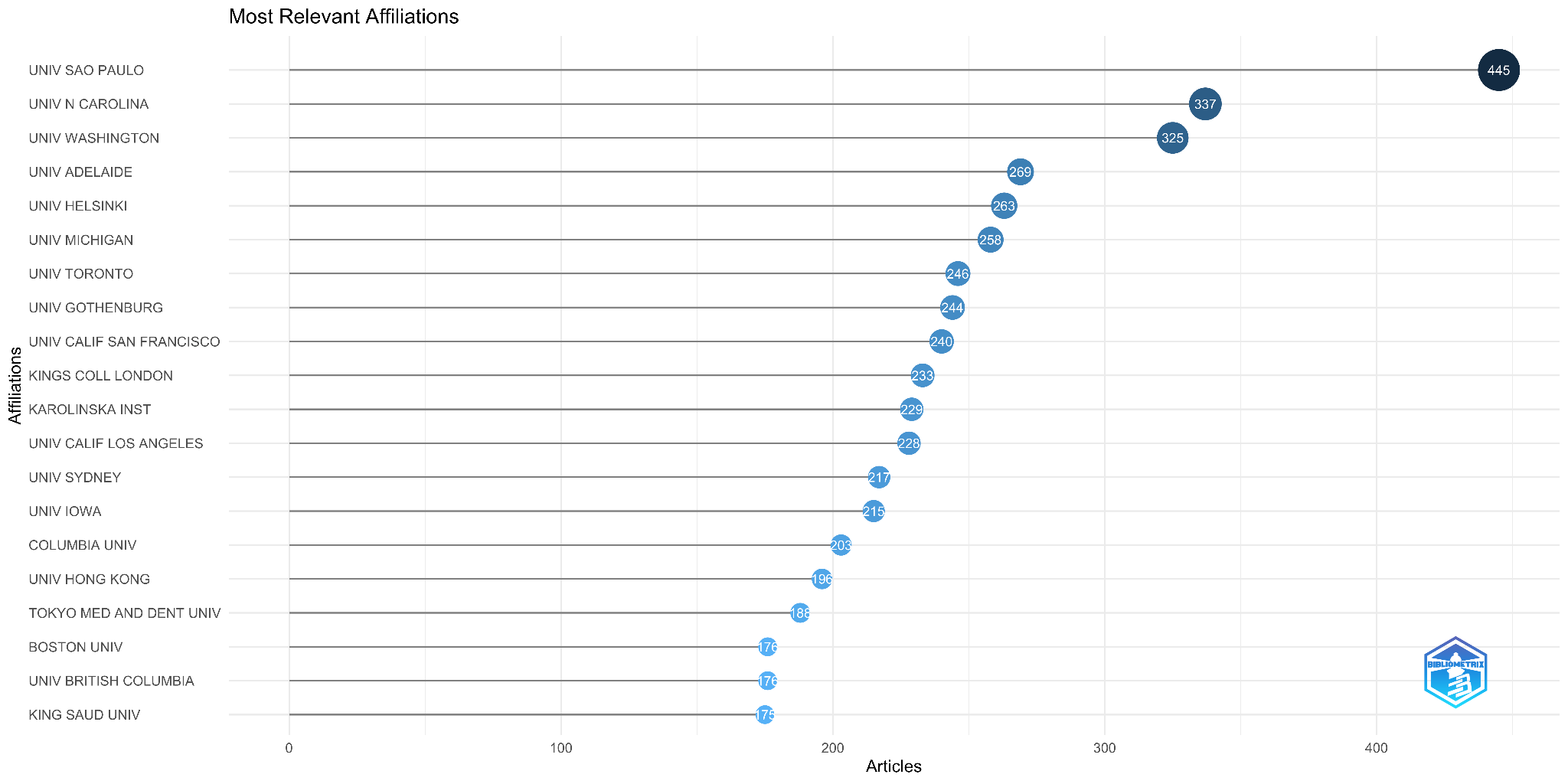


**Author’s keyword adjustment**

**Removed**

| oral complications,oral health,risk factors,dentistry,knowledge,dental,prevalence,qualitative research,questionnaire,risk factor,treatment,survey,oral,cross-sectional study,diagnosis,incidence,treatment needs,reliability,clinical trial,cohort study,longitudinal study,validity,practice,cross-sectional studies,complications,intervention,periodontal,risk assessment,assessment,longitudinal studies,randomized controlled trial,patients,qualitative,etiology,health,cohort studies,perception,questionnaires,treatment outcome,meta-analysis,treatment need,surveys and questionnaires,risk indicators,validation,outcomes,perceptions,case-control study,follow-up studies,longitudinal,multilevel analysis,oral diagnosis,qualitative study,follow-up,late effects,outcome,practices,survival analysis,association,cross-sectional,pathology,prognosis,risk,cohort,dental patients,factor analysis,human,review,systematic review,focus group,associated factors,evaluation,grounded theory,logistic models,logistic regression,randomized clinical trial,recruitment,severity,clinical trials,collaboration,community-based participatory research,complication,mercury,oral findings,polymerase chain reaction,predictors,randomised controlled trial,real-time polymerase chain reaction,success,teaching,trends,dental problems,case-control studies,clinical study,decision making,experiences,population study,professional role,prospective cohort study,referral,regression analysis,retrospective study,structural equation modelling,syndrome,validation studies |
| --- |

**Merged***

| access,access to care,access to health care,accessibility  access to dental care,access to oral health care  adolescents,adolescent,adolescenceyoung adults  adults,adult  alzheimer's disease,alzheimer disease  anaesthesia,anesthesia  anorexia,anorexia nervosa  anticoagulation,anticoagulants,anticoagulants,oral anticoagulants,anticoagulant therapy  attachment loss,periodontal attachment loss,clinical attachment loss,loss of attachment  attitudes,attitude  autism,autism spectrum disorder,autism spectrum disorders,autistic disorder,autistic spectrum disorder  behaviour,behavior  biomarkers,biomarker,biological markers  bisphosphonates,bisphosphonate  bmi,body mass index  bone metastasis,bone metastases  breast cancer,breast neoplasms  bronj, bisphosphonate-related osteonecrosis of the jaw  buffering capacity,buffer capacity  bulimia nervosa,bulimia  calculus,dental calculus  cancer treatment,cancer therapy  candida,candida albicans,candida spp  cardiovascular diseases,cardiovascular disease  caregivers,caregiver  caries,dental caries,tooth decay,dental decay,decayed  children,child  chronic diseases,chronic disease  cleft,cleft palate,cleft lip and palate,cleft lip,cleft lip/palate  cognitions,cognition,cognitive function  cognitive impairment,cognitive dysfunction  complete dentures,complete denture  computed tomography,ct  cytokines,cytokine  dental care,oral care  dental diseases,dental disease  dental erosion,erosive tooth wear  dental health surveys,dental health survey  dental hygienists,dental hygienists  dental services,  dental services utilization,dental utilization  dental visits,dental visit  dentists,dentist  dentures,denture  developmental disabilities,developmental disability  diabetes,diabetes mellitus  diet, dietary intake,dietary habits  disabilities,disability  disparaties,disparity  dmft,dmf index,dmft index,dmf  down syndrome,down's syndrome  drug abuse,substance abuse,drug addiction  dry mouth,oral dryness  edentulism,edentulous,edentulousness  elderly,older adults,aged,older people,aging,ageing,elderly people,elders,elderly patients,the elderly,geriatric population,elderly population,older,older adult,older persons,elder,seniors  enamel,dental enamel  endodontics,endodontic  ethnicity,race,ethnic groups  extraction,tooth extraction,dental extraction,dental extractions  fluorides,fluoride  fluorosis,dental fluorosis  free flibula flap, fibula free flap  gender,sex  general anaesthesia, general anesthesia  geriatrics,geriatric  gingivitis,gingival inflammation  glycaemic control,glycemic control  head and neck cancer,head and neck neoplasms  health behaviors,health behavior  health disparities,health inequalities  health literacy,health knowledge  hearing impairment,hearing loss  hispanics,hispanic  hiv,hiv infection,hiv/aids,human immunodeficiency virus,hiv infections  homeless,homeless persons,homelessness  immigrants,immigrant,immigration,emigrants and immigrants,migrants,migrant  implants,dental implants,dental implant,implant,dental implantation,implantology  inequalities,inequality  institutionalised elderly,institutionalized elderly  intellectual disabilities,intellectual disability  jaw,jaws  mandibular advancement devices,mandibular advancement device,mandibular advancement appliance  mandibular reconstruction,mandible reconstruction  masticatory performance,chewing ability,masticatory performance,masticatory function,masticatory ability  medications,medication  mental disorders,mental illness  microbiome,microbiota  mronj,medication-related osteonecrosis of the jaw  nursing homes,nursing home  obstructive sleep apnoea,obstructive sleep apnea,obstructive sleep apnea syndrome  occlusion,dental occlusion  ohip-14,oral health impact profile,ohip,oral health impact profile-14  ohrqol,oral health-related quality of life,oral health related quality of life,oral health quality of life  oral appliances,oral appliance,oral appliance therapy  oral cancer,mouth neoplasms,oral cavity cancer,mouth neoplasm  oral diseases,oral disease  oral health behaviours,oral health behavior,oral health behaviors,oral health behaviour  oral health status,oral status  oral hygiene,dental hygiene  oral infections,oral infection  oral lesions,oral lesion  oral manifestations,oral manifestation  oral microbiome,oral microbiota,oral microflora  oral mucosa,mouth mucosa  oral surgery,oral surgical procedures  osteonecrosis of the jaw,jaw osteonecrosis,osteonecrosis of the jaws  paediatric dentistry,pediatric dentistry  paediatrics,pediatrics,pediatric,paediatric  periodontal therapy,periodontal treatment  periodontitis,periodontal diseases,periodontal inflammation  permanent dentition,permanent teeth  personality traits,personality  physical function,physical performance  pregnancy,pregnant women  preschool children,preschool  primary care,primary health care  primary dentition,primary teeth  prison,prisoners  prosthesis,prosthetic rehabilitation,prosthetic treatment  psychometrics,psychometric properties  radiotherapy,radiation therapy  rare diseases,rare disease  renal transplant,renal transplantation  salivary flow,salivary flow rate  school,schools  school children,schoolchildren  sleep apnoea,sleep apnea,sleep disordered breathing,sleep-disordered breathing,sleep apnea syndromes  social inequality,social inequalities  sociodemographic factors,socio-demographic factors  socioeconomic factors,socio-economic factors  socioeconomic status,socio-economic status  special needs,special care,special health care needs  special needs dentistry,special care dentistry  surgery,surgical treatment  swallowing,deglutition  systemic diseases,systemic disease  teeth,tooth  tmd,temporomandibular disorders,temporomandibular disorder,temporomandibular joint disorders  smoking,tobacco use,tobacco smoking,tobacco  toothbrushing,tooth brushing  type 1 diabetes,type 1 diabetes mellitus  type 2 diabetes,diabetes mellitus type 2,type 2 diabetes mellitus  usa,united states  utilisation,utilization |
| --- |

*subsequent keywords merged into the first keyword of the row
